# Gray matter correlates of creative potential: A latent variable voxel-based morphometry study

**DOI:** 10.1016/j.neuroimage.2015.02.002

**Published:** 2015-05-01

**Authors:** Emanuel Jauk, Aljoscha C. Neubauer, Beate Dunst, Andreas Fink, Mathias Benedek

**Affiliations:** Department of Psychology, University of Graz, BioTechMed-Graz, Austria

**Keywords:** Creativity, Voxel-based morphometry (VBM), Gray matter, Precuneus, Intelligence, Openness to experience

## Abstract

There is increasing research interest in the structural and functional brain correlates underlying creative potential. Recent investigations found that interindividual differences in creative potential relate to volumetric differences in brain regions belonging to the default mode network, such as the precuneus. Yet, the complex interplay between creative potential, intelligence, and personality traits and their respective neural bases is still under debate. We investigated regional gray matter volume (rGMV) differences that can be associated with creative potential in a heterogeneous sample of *N* = 135 individuals using voxel-based morphometry (VBM). By means of latent variable modeling and consideration of recent psychometric advancements in creativity research, we sought to disentangle the effects of ideational originality and fluency as two independent indicators of creative potential. Intelligence and openness to experience were considered as common covariates of creative potential. The results confirmed and extended previous research: rGMV in the precuneus was associated with ideational originality, but not with ideational fluency. In addition, we found ideational originality to be correlated with rGMV in the caudate nucleus. The results indicate that the ability to produce original ideas is tied to default-mode as well as dopaminergic structures. These structural brain correlates of ideational originality were apparent throughout the whole range of intellectual ability and thus not moderated by intelligence. In contrast, structural correlates of ideational fluency, a quantitative marker of creative potential, were observed only in lower intelligent individuals in the cuneus/lingual gyrus.

## Highlights

• We studied gray matter correlates of creativity by means of voxel-based morphometry.
• Latent variables were used as predictors of regional gray matter volume.
• Creative potential was measured by means of ideational originality and fluency.
• Originality was correlated to gray matter in the precuneus and nucleus caudatus.
• Fluency was correlated to cuneus gray matter, but only in lower IQ individuals.

## Introduction

Creativity has become a topic of increasing interest to cognitive and neuroscientific psychology (Dietrich and Kanso, 2010). In a world changing more rapidly than ever before, the ability to come up with creative new ideas is of extraordinary importance to cultural development and the progress of human civilization. While creativity has long been considered a dark and nebulous phenomenon that is reserved to eminent geniuses and cannot be subject to population-based studies, an emerging line of research triggered by Guilford's (1950) influential ideas has begun to demystify both the creative process and the creative person. Creative idea generation is now viewed as a common cognitive process that is of relevance to many areas of everyday life (e.g. Silvia et al., 2014) and creative potential is known to reflect a normally distributed trait just as any other mental ability (Eysenck, 1995). Yet, the neuroscientific investigation of creativity is still in its infancy and much work needs to be done in order to gain a deeper understanding of the creative brain (Abraham, 2013; Arden et al., 2010; Dietrich and Kanso, 2010; Fink and Benedek, 2014a,b; Sawyer, 2011).

### Creative potential as a cognitive marker of real-life creativity

Creative potential is usually defined as the ability to produce something *novel* and *useful*, also known as the “standard definition of creativity” (Runco and Jaeger, 2012, p. 92; see also Barron, 1955; Stein, 1953). This ability can be assessed by means of divergent thinking tests, which have proved to be reliable and valid indicators of a person's creative potential (e.g., Benedek et al., 2014b; Benedek et al., 2013; Runco and Acar, 2012; Silvia et al., 2008). A common divergent thinking task is the alternate uses task that asks participants to find many uncommon and creative uses for objects of daily use (e.g., a can). Individuals differ with respect to their abilities to produce a high *quantity* (ideational fluency) and a high *quality* (ideational originality) of ideas in these tasks. Both, quantitative and qualitative indicators were found to have predictive validity with respect to real-life creative accomplishments across different domains including music, arts, or science (Jauk et al., 2014). Creative potential is known to be associated with openness to experience (Batey and Furnham, 2006; Feist, 1998, 2010; Nusbaum and Silvia, 2011a) and intelligence (Batey and Furnham, 2006; Kim, 2005; Nusbaum and Silvia, 2011b). Moreover, the creativity–intelligence relationship was found to be moderated by the intelligence level, which is referred to as *threshold effect* (Guilford, 1967). Intelligence may be relevant for creative potential up to an above-average IQ but loses its impact thereafter (Jauk et al., 2013; Karwowski and Gralewski, 2013). Thus, intelligence can be conceived a cognitive prerequisite of creative potential. In other words, above-average intelligence forms a necessary, but not sufficient condition for high creative potential. Openness, in contrast, may influence creativity even at fairly high levels of IQ (Jauk et al., 2013).

### Functional brain mechanisms underlying creative idea generation

EEG studies of divergent thinking processes generally show that EEG alpha power is indicative of creative idea generation (Fink and Benedek, 2014a). Increased alpha power is assumed to reflect a state of internally focused attention that facilitates processes of semantic search and imagination involved in the generation of new ideas (Benedek et al., 2011; Benedek et al., 2014d). Functional MRI studies of divergent thinking revealed, among others, activation in the left inferior and superior frontal gyri and inferior parietal regions such as the angular gyrus (Abraham et al., 2012; Benedek et al., 2014c; Fink et al., 2009; for a recent meta-analysis, see Gonen-Yaacovi et al., 2013). These results point to a central role of (prefrontal) executive as well as (parietal) memory-related processes that are known to be crucial for the fluent production of novel ideas from behavioral research (Beaty et al., 2014b; Benedek et al., 2012; Nusbaum and Silvia, 2011b; Silvia et al., 2013). Studies using research paradigms other than divergent thinking, however, could not yet reveal consistent results, which is most likely due to the variety of employed tasks and measures (Arden et al., 2010; Dietrich and Kanso, 2010; Fink and Benedek, 2014a; Sawyer, 2011).

### Brain structural correlates of creative potential

Although there exists converging evidence on the functional mechanisms underlying creative idea generation, it remains an intriguing question whether individual differences in creative potential relate to differences in brain *structure*. In one of the first studies, Jung et al. (2010) found negative correlations between creative potential (originality) and cortical thickness in several, mostly right-hemispheric regions including posterior areas such as the cuneus and the inferior parietal cortex; only one positive association was observed in the right posterior cingulate cortex. Takeuchi et al. (2010) used voxel-based morphometry (VBM) to identify volumetric differences related to creative potential. They found positive correlations between regional gray matter volume (rGMV) and creative potential scores in the right dorsolateral prefrontal cortex, bilateral striate, a cluster including midbrain structures, and regions in the precuneus; no negative relationships were reported in this study. Both, Jung et al. (2010) and Takeuchi et al. (2010) used participant's sex, age, and general intelligence as covariates in their regression models to control for possible influences of these variables. However, the results of the different structural parameters (cortical thickness and rGMV) cannot be directly compared to each other. A recent study reported significant correlations between verbal creative potential and rGMV in the bilateral inferior frontal gyri (Zhu et al., 2013). The effects were also controlled for sex, age, general intelligence, and additionally total gray matter volume. Fink et al. (2014a) investigated regional gray matter density correlates of different indicators of verbal creative potential, namely ideational fluency and ideational originality as well as a combined fluency/flexibility score (i.e., number of responses and number of different categories these responses belong to). They found that ideational originality correlated positively with density in the right cuneus while the fluency/flexibility score showed correlations in the right precuneus and cuneus. No significant effects were observed for the pure fluency score. Similar to Zhu et al. (2013), participant's age, sex, general intelligence, and total intracranial volume were considered as covariates. Similarly, Kühn et al. (2014) found structural correlates of ideational originality in the precuneus (albeit in the left hemisphere), the ventromedial prefrontal cortex, and also the left insula and the right temporo-parietal junction. Another study examining visual creative potential reported associations with right-parietal rGMV (Gansler et al., 2011).

As Jung et al. (2013) conclude in their recent review, one of the most striking findings across morphometric studies of creative potential is that many of the regions repeatedly reported belong to the default mode network (DMN; Gusnard and Raichle, 2001; Raichle and Snyder, 2007). Three out of four studies investigating verbal creative potential by means of VBM reported positive associations between indicators of creativity and brain structre in the precuneus. The precuneus was also found to be *functionally* involved in divergent thinking (Benedek et al., 2014c; Fink et al., 2010, 2012) and metaphor generation (Benedek et al., 2014a). Specifically, it was observed that the precuneus, which – as part of the DMN – is usually deactivated during cognitive tasks, shows weaker deactivation in high- as compared to low-schizotypic individuals during creative cognition (Fink et al., 2014b). In a similar vein, highly creative individuals show reduced deactivation in the precuneus during a working memory task (Takeuchi et al., 2011). These findings conform to the notion that the precuneus is involved in internally guided attention (Cavanna and Trimble, 2006); a process closely associated with creativity (Fink and Benedek, 2014a).

While intelligence was considered a covariate of no interest in most of the studies reported above, one study explicitly addressed the role of intelligence as a moderator of the brain–creativity-relationship: Jung et al. (2009) conducted a magnetic resonance spectroscopy study and found that IQ level moderates the relationship between creative potential and the concentration of *N*-acetyl-aspartate (NAA), a marker of neuronal integrity. The authors interpret their findings in terms of increased left-hemispheric functioning in higher intelligent people, which might facilitate access to left-hemispheric semantic networks. To date, however, no study examined whether intelligence may also moderate the relationship between creative potential and brain *structure* in terms of rGMV.

### The present research

This study investigates rGMV correlates of creative potential by means of voxel-based morphometry. Creative potential can be assessed by different indicators. We used scores of ideational fluency and originality in order to account for both quantitative as well as qualitative indicators of creative potential. Moreover, these measures were shown to have discriminant validity given an adequate scoring that avoids the confounding influence of fluency (Benedek et al., 2013; Jauk et al., 2014; Silvia et al., 2008).

While VBM is considered a highly reliable method (Jung et al., 2013), the tests commonly used to assess creative potential sometimes show low reliability (Arden et al., 2010; Dietrich and Kanso, 2010). Therefore, we use structural equation modeling (SEM) to obtain latent factors of ideational fluency and originality based on an extended set of six divergent thinking tasks. SEM allows accounting for measurement error in observed variables in order to obtain “true” scores of the underlying psychological constructs. Latent scores help to overcome common pitfalls in psychometric research (cf. Silvia, 2008) and can be used as powerful predictors in neuroimaging studies (cf. Colom et al., 2013). Finally, we included intelligence and openness to experience as covariates since they are known to be correlated with creative potential. Considering the influence of these relevant covariates allows determining gray matter effects that are specific to creative potential.

Given the often inconsistent findings regarding the neuroscience of creativity (Arden et al., 2010; Dietrich and Kanso, 2010) and of neuroscientific findings in general (Uttal, 2012), an attempt of replication and extension of previous findings using state-of-the-art methods is considered a powerful and necessary means toward establishing dependable empirical evidence. Taking into account evidence from behavioral and neuroimaging studies of creative potential, we expected differences in rGMV in prefrontal regions that are relevant to executive functioning (Takeuchi et al., 2010; Zhu et al., 2013) and parieto-occipital regions (including the precuneus and cuneus; Fink et al., 2014a; Takeuchi et al., 2010). Additionally, we investigate whether correlations between rGMV and creative potential are moderated by the intelligence level. Considering behavioral findings on the threshold hypothesis on the relationship between creative potential and intelligence, we hypothesized that creative potential might be more tied to brain structure in subjects of lower IQ and weakens as IQ level increases.

## Method

### Participants

Participants were recruited via a local newspaper and the university's mailing lists. They took part in a larger research project^1^ and were screened for a variety of psychological variables (see also Jauk et al., 2013). Structural MRI scans were obtained from 141 individuals. Two participants were identified as multivariate outliers with respect to the psychological variables under study (Mahalanobis distance *p* < .001) and further four were excluded due to low image covariance (see below) leading to a final sample of *N* = 135. All participants were right-handed, had German as mother tongue, and reported no prior neurological and/or mental disorders. Participant's intelligence level was about half a standard deviation above the general population mean (IQ = 108.70) and had a fairly representative distribution (*SD* = 13.54). Table 1 displays detailed sample characteristics. The study was approved by the Ethics Committee of the University of Graz.

### Psychometric tests

Psychometric measures were acquired in the course of a larger research project previously reported (Dunst et al., 2014; Jauk et al., 2013, 2014). All variables used in the present study reflect factor scores extracted from the structural equation model (model A) presented in Jauk et al. (2014) using Mplus 7 (Muthén & Muthén, Los Angeles, CA). The scoring and modeling conforms exactly to Jauk et al. (2014) and is thus only briefly presented here.

*Creative potential* (*fluency* and *originality*) was assessed by means of six independent divergent thinking tasks; three alternate uses (AU) and three instances (IN) tasks. Ideational fluency was defined as the number of ideas given in each task. Ideational originality was assessed by means of subjective top-3 scoring, which reflects the rated creativity of the three best ideas within each task. This scoring overcomes the typically high confounding of fluency and originality indicators (Benedek et al., 2013). Mean interrater reliabilities were *ICC* = .80 in the AU tasks and *ICC* = .69 in the IN tasks. Using the resulting six task-wise fluency and originality measures as indicators, we obtained two latent variables (*fluency* and *originality*) by means of higher-order confirmatory factor analysis (CFA).

*General intelligence* was measured using three subtests of the Intelligence Structure Battery (Intelligenz-Struktur-Batterie, INSBAT; Arendasy et al., 2009), which is theoretically grounded on the Cattell–Horn–Carroll model of intelligence (for an overview, see McGrew, 2009). The three computer-based tests reflect facets of *gf* including figural inductive reasoning, verbal short-term memory, and arithmetic flexibility. The INSBAT is based on item response theory (IRT) and allows for tailored testing. All subtests conform to the 1PL Rasch model and display good construct and criterion validities (for an overview, see Arendasy et al., 2009). The minimum individual reliability for each scale was set to α = .60.

*Openness to experience* was assessed with the Big Five Structure Inventory (Arendasy et al., 2011). The test is based on IRT and was shown to have high reliability and validity (Arendasy et al., 2011).

### MRI data acquisition and processing

Whole brain imaging was performed on two different 3 T MRI scanners. From the behaviorally valid sample of 139 persons, 40 (27 female) were examined in scanner 1 which was a 3 T Tim Trio system (Siemens Medical Systems, Erlangen, Germany). The remaining 99 persons (57 female) were examined in scanner 2, which was a 3 T Siemens Magnetom Skyra system (Siemens Medical Systems, Erlangen, Germany). On both scanners, we used a 3D-MPRAGE sequence (176 slices per slab, FOV = 256 mm, TR = 1560.00 ms, TE = 2.07 ms, voxel size = 1 mm isotropic) with a 32-channel head coil.

We performed MRI data analyses using the VBM8 toolbox (Gaser, C., http://dbm.neuro.uni-jena.de/vbm/, revision 435). VBM8 is based on SPM8 software (Wellcome Department of Imaging Neuroscience, London, UK) running on Matlab version 7.12 (MathWorks Inc., Natick, MA). Preprocessing steps included bias-field correction and segmentation into gray matter (GM), white matter (WM), and cerebrospinal fluid (CSF). Segmented images were registered to standard Montreal Neurological Institute (MNI) space using the high-dimensional Dartel approach (Ashburner and Friston, 2005). Modulation was performed using the “non-linear only” option in VBM8 which corrects for different individual brain sizes. The resulting images can be interpreted in terms of relative volume without any further statistical correlation for brain size (Kurth et al., 2010). Finally, GM images were smoothed with a Gaussian kernel of 10 mm^3^ FWHM.

A quality check of GM images was performed using the “check sample homogeneity using covariance” — function implemented in VBM8. Four participants (all male, examined in scanner 2) were excluded due to covariance less than 2 *SD* below the sample mean leading to a final sample of *N* = 135 participants. The resulting mean GM image covariance was .97.

### Analysis plan

To identify individual differences in rGMV related to creative potential, we used voxelwise whole-brain multiple regression analyses implemented in SPM8. As in similar previous studies (Fink et al., 2014a; Takeuchi et al., 2010; Zhu et al., 2013), each model included the variables *sex*, *age*, and *intelligence* as covariates (individual brain size was not included; see above). Additionally, we also included *openness to experience* as a covariate, as openness is consistently found to be related to both creative potential (Batey and Furnham, 2006; Feist, 1998) and intelligence (Ackerman and Heggestad, 1997; DeYoung, 2011). Finally, the variable *scanner* (binary-coded as scanner 1/2) was included to account for possible sensitivity differences between the two MRI scanners. It has been shown that modeling different scanners can effectively account for scanner variance in VBM and does not affect contrasts of interest (Stonnington et al., 2008). The minimum gray matter density was set to .10. Like in Fink et al. (2014a), all reported findings were corrected for multiple comparisons by means of cluster size correction as implemented in the AFNI suite (AFNI program 3dClustSim, the successor of AlphaSim; Cox, 1996). 3dClustSim simulates a random field of FWHM-smoothed noise and computes the minimum size of non-noise clusters at given voxelwise and cluster-level *p*-thresholds. We used voxelwise *p* < .001 and cluster-level *p* < .05 and performed 3dClustSim correction separately for each multiple regression model using mean smoothness parameters obtained from the respective residual image (which leads to slightly varying cluster size [k] — thresholds for each model).

In order to investigate whether intelligence moderates the relationship between indicators of creative potential and rGMV, we split the sample at the latent intelligence median and performed region of interest (ROI) analyses for areas of significant correlation in the full sample using the MarsBaR 0.43 toolbox (Brett et al., 2002). Unlike whole-brain analyses, ROI analyses yield single regression weights for each area of investigation that can then be compared across groups of lower and higher intelligence (which is the prevalent approach to investigate the threshold hypothesis). The factors included in these models were *originality*, *fluency*, *intelligence* (within-groups), *openness*, *age*, *sex*, and *scanner*. Additionally, we performed separate voxelwise whole-brain analyses for the subsamples of lower and higher IQ in order to check for effects in areas other than the ROIs.

## Results

### Descriptive statistics and intercorrelations

Table 1 displays descriptive statistics and intercorrelations of the study variables. We observed a high latent correlation between ideational originality and intelligence. Ideational fluency was not related to intelligence, and the correlation between fluency and originality just reached statistical significance. Both, ideational originality and ideational fluency, were significantly related to openness, but intelligence was not. Interestingly, intelligence and ideational originality were moderately related to total GM volume (and thus also to TIV). Age had slight negative associations with cognitive measures, and males did on average better on tests of originality and intelligence. Age was correlated with scanner, indicating that the sample tested in scanner 1 was somewhat younger; however, none of the other measures, including global tissue volumes, were correlated with scanner.

### VBM results

#### Voxelwise whole-brain analysis

**Fig. 1:**
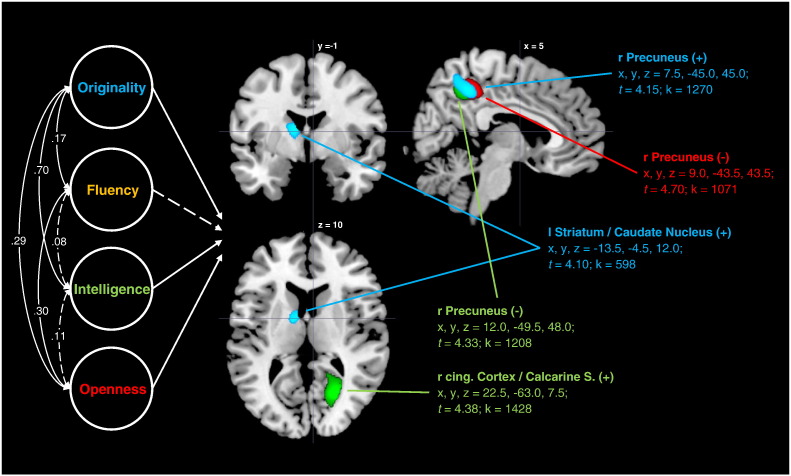
Regions of correlation between rGMV and variables under study in the full sample. Parentheses denote the direction of correlation.

Fig. 1 displays the results of the voxelwise whole-brain multiple regression analysis. Given a cluster size threshold of k > 524 voxels, we observed significant positive associations between ideational originality and rGMV in the precuneus (x, y, z = 7.5, − 45.0, 45.0; *t* = 4.15; k = 1270) and in the left striatum (x, y, z = − 13.5, − 4.5, 12.0; *t* = 4.10; k = 598). While the striatal effect was observed only in the left hemisphere, the effect in the precuneus was lateralized to the right hemisphere (with 706 voxels being in the right and 476 in the left precuneus). No negative effects were observed for ideational originality. For ideational fluency, no significant effects were observed. For intelligence, we observed a positive effect in the right posterior cingulate cortex/calcarine sulcus (x, y, z = 22.5, − 63.0, 7.5; *t* = 4.38; k = 1428). A negative association between intelligence and rGMV was observed in the (predominantly right) precuneus (x, y, z = 12.0, − 49.5, 48.0; *t* =  4.33; k = 1208). Similarly, openness showed a negative association with rGMV in the precuneus (x, y, z = 9.0, − 43.5, 43.5; *t* =  4.70; k = 1071).

#### ROI analyses (lower vs. higher intelligent individuals)

In order to investigate whether the relationship between rGMV and ideational originality may be moderated by participant's intelligence level, we conducted separate multiple regression models for the prediction of mean rGMV in the regions reported above. We split the sample at the median of the latent intelligence score (corresponding to an observed IQ of 107.00; Lower IQ group: *M* = 98.58 (SD = 7.64), *n* = 67; Higher IQ group: *M* = 118.85 (*SD* = 10.26), *n* = 67). ROIs were obtained from the results of the whole-brain analysis (binary masks of contrast estimates) presented in the preceding paragraph. As shown in Table 2, regression weights for originality are consistent across both groups and thus not moderated by the general intelligence level.

#### Voxelwise whole-brain analyses (lower vs. higher intelligent individuals)

**Fig. 2:**
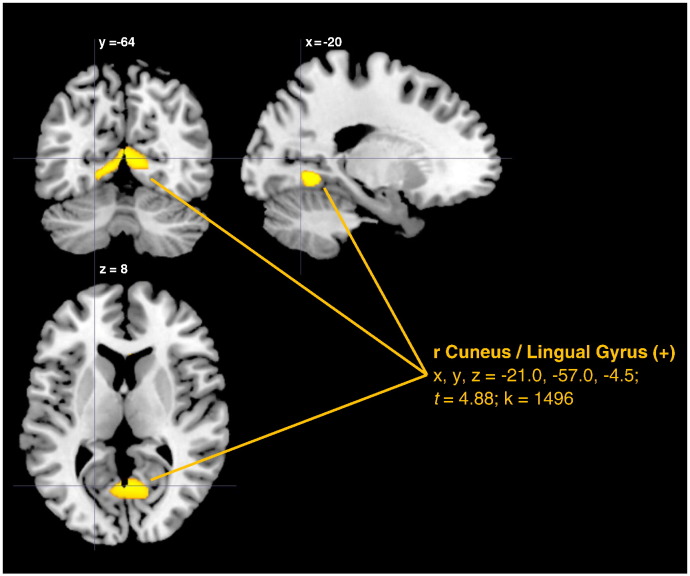
Regions of positive correlation between rGMV and ideational fluency in lower intelligent individuals.

In lower intelligent individuals, no significant (cluster size k > 542) positive or negative effects were observed for ideational originality. For ideational fluency, we observed a significant positive effect in the bilateral cuneus/lingual gyrus (x, y, z = − 21.0, − 57.0, − 4.5; *t* = 4.88; k = 1496; Fig. 2). Like in the full sample, intelligence was positively associated with rGMV in the right calcarine sulcus (x, y, z = 21.0, − 58.5, 3.0; *t* = 4.33; k = 1367) and also negatively associated to rGMV in a cluster extending from the brainstem to the left thalamus (x, y, z = 0.0, − 18.0, 19.5; *t* =  4.08; k = 738). Openness, in contrast, was negatively correlated with a cluster in the right middle temporal lobe (x, y, z = 45.0, − 48.0, 1.5; *t* =  4.73; k = 827).

In higher intelligent individuals, no significant positive or negative effects (k > 554) were observed for any of the involved measures.

## Discussion

This study investigated brain structural correlates of creative potential. We used voxel-based morphometry to unveil interindividual differences in regional gray matter volume that can be associated with ideational originality and ideational fluency, two major psychometric markers of creative potential. By means of employing the subjective top-scoring method (Benedek et al., 2013; Silvia et al., 2008) and the use of latent variable modeling, we obtained independent, reliable, and valid indicators of ideational originality and ideational fluency. Intelligence and openness to experience were considered as covariates as they are known to share substantial amounts of variance with creative potential (cf. Batey and Furnham, 2006; Jauk et al., 2014). We hypothesized that creative potential may be correlated with rGMV in DMN regions such as the precuneus, which is probably the most consistent finding across structural (Fink et al., 2014a; Kühn et al., 2014; Takeuchi et al., 2010) and functional (Benedek et al., 2014a; Fink et al., 2010, 2012) imaging studies of creativity. Moreover, creative potential was hypothesized to relate to rGMV in prefrontal regions including the inferior frontal gyrus or the ventromedial prefrontal cortex (Kühn et al., 2014; Takeuchi et al., 2010; Zhu et al., 2013).

Our analyses confirmed the first hypothesis as we found rGMV in the precuneus to be significantly correlated with ideational originality. The positive association of creative potential and gray matter volume in the precuneus thus was replicated in a large sample using latent measures of creative potential. This effect was specific to ideational originality but not fluency, suggesting that the precuneus is associated with individual differences in the ability to generate *creative* ideas rather than large amounts of ideas. Moreover, the effect cannot be attributed to other correlated traits such as intelligence or openness (which were included as covariates in the analysis) and was homogenous across groups of lower and higher intelligence. This result hence confirms the relevance of the precuneus for creative potential and adds to the clarification of the specificity of this finding.

We did, however, not observe any prefrontal effects related to ideational originality or ideational fluency. Instead, we found ideational originality to be significantly correlated to rGMV in regions of the left striatum, particularly in the caudate nucleus body, a result also observed by Takeuchi et al. (2010). In the following sections we try to elaborate on the roles of the precuneus and left striatal regions for creative potential.

### The precuneus as a neural substrate of ideational originality

Due to its hidden location in the brain, the precuneus has received little research attention until the dawn of functional imaging (Cavanna and Trimble, 2006). Recent studies point to a pivotal role of the precuneus in general conscious awareness, as it was found, for instance, that metabolism in the precuneus directly relates to the degree of anesthesia (Vogt and Laureys, 2005). During wake resting state, the precuneus, along with the adjacent posterior cingulate cortex, shows markedly higher metabolism than other brain regions (Raichle et al., 2001). As soon as individuals perform cognitively demanding tasks that require an external focus of attention, however, activation in the precuneus – as a part of the DMN – decreases and other networks come into play (Buckner et al., 2008). That is, externally directed attention effectively suppresses the brain's self-referential processing and paves the way for task-specific activation. The observation that the DMN is usually deactivated during cognitive paradigms (which mostly demand external direction of attention) has led to the DMN being labeled a “task negative network” (Fox et al., 2005). As Leech and Sharp (2014) point out, however, “this is misleading as increased DMN activity is observed in many situations where attention is internally directed” (p. 15; for a detailed discussion, see Spreng, 2012). In line with this notion, functional imaging studies that rely on internal attention report active involvement of the precuneus in processes such as episodic memory retrieval or self-processing operations (Cavanna and Trimble, 2006). More generally, DMN-activation can be observed during various kinds of loose self-referential thought or “mind wandering” (Spreng, 2012; see also Anticevic et al., 2012). These functions may directly relate to creativity, as creative cognition has long been hypothesized to draw strongly upon primary process cognition (Kris, 1952), i.e. autonomous and associative processing with an internal focus of attention that goes along with increased EEG alpha power (Fink and Benedek, 2014a). In line with this, creative people were found to report more fantasy activity and remember their dreams better (Martindale, 1999). Mind wandering was also found to directly enhance creative thinking (Baird et al., 2012), although it may be detrimental to intelligence-related demands (Mooneyham and Schooler, 2013). Takeuchi et al. (2011) found that people with higher creative potential “failed” to deactivate the precuneus during a working memory task, which might indicate that they disengage default mode processes to a lesser extent during cognitively demanding tasks. In a similar vein, people scoring high on schizotypy (a trait related to creative potential; Eysenck, 1995; see also Batey and Furnham, 2006) showed reduced deactivation in the precuneus during a divergent thinking task (Fink et al., 2014b). Recently, we found stronger involvement of the precuneus during a creative cognition task (metaphor generation) as compared to a related but non-creative control task (synonym generation; Benedek et al., 2014a). In a similar vein, Bar et al. (2007) demonstrated that the presentation of objects that induce a rich associative process elicit stronger neural responses in the precuneus than objects with a weak associative context. Moreover, the precuneus was found to act as a hub within the DMN and is functionally connected with the left inferior parietal lobe and the dorsal and ventral medial prefrontal cortex (Fransson and Marrelec, 2008). The inferior parietal lobe, in turn, is associated with the production of new ideas (i.e., ideas that were spontaneously created rather than recalled from memory) during divergent thinking (Benedek et al., 2014c). Finally, we recently found that highly creative people show increased resting state connectivity between prefrontal-executive and DMN regions such as the posterior cingulate cortex and the adjacent precuneus (Beaty et al., 2014a).

Summing up, there exists strong evidence that the precuneus – as part of the DMN – plays a central role in creative cognition. Both, functional and structural imaging studies found involvement of the precuneus in creative cognition on within- and between-subjects levels. While activation (or reduced deactivation) of the precuneus during functional imaging is thought to reflect a state of internally focused attention, mind wandering, or self-referential thought, the association of between-subjects variability in precuneus rGMV with individual creative potential indicates that this region is not only indicative of creativity as a *state* but also as a *trait*: People who have habitually higher creative potential show increased rGMV in the precuneus. Higher precuneus rGMV could thus be thought to facilitate divergent thinking by means of an increased proneness to an internal focus of attention and primary processes such as mind wandering. Of course, brain structure may either be a cause or a consequence (in terms of neural plasticity) of these behavioral phenomena — longitudinal research would be needed to clarify this question.

Interestingly, intelligence and openness to experience were negatively correlated with rGMV in the precuneus (although creative potential was positively related). In post-hoc analyses, we closely examined our data using hierarchical regression models to rule out the possibility of complex suppression effects. We found that the same tendencies were also apparent when we entered the single variables into one regression model at a time. Thus, it seems that while creative potential and intelligence as well as openness are positively related on a behavioral level, there seems to a trade-off on the neurostructural level. This might at least partially be explained along the line presented above: While primary processes such as mind wandering are generally beneficial to creative thought, they can be detrimental to intelligence-related demands (Mooneyham and Schooler, 2013). Thus, highly creative people (who are, given the substantial correlation, also of higher intelligence) may devote some of their gray matter to primary process cognition while – at the same time – this primary processes may hinder convergent thinking ability to a certain extent. To this end, it should be noted that the structures of positive and negative correlation were only partially overlapping. Given that the precuneus was found to comprise functionally specific subdivisions (Margulies et al., 2009), further functional imaging studies could help to clarify the complex interplay between ideational originality, openness, and intelligence within the precuneus.

### Striatal contributions to ideational originality

In addition to the cluster in the precuneus, we found rGMV correlates of ideational originality in the left striatum including parts of the caudate nucleus. This result is similar to the findings of Takeuchi et al. (2010). Specifically, they found rGMV in the bilateral caudate nucleus and substantia nigra to vary as a function of creative potential. The authors argue that these regions are part of the dopaminergic system, and thus, interindividual differences in creative potential may relate to dopaminergic regulation. Dopamine is generally associated with novelty-seeking (Flaherty, 2005) and was labeled the “neuromodulator of exploration” with respect to personality (DeYoung, 2013). Given that novelty-seeking is a driving force of creativity (Panksepp and Biven, 2007), there is converging direct and indirect evidence for the involvement of the dopaminergic system in creativity (Beversdorf, 2013). It was recently found that genetic variations associated with dopaminergic functioning predict ideational fluency and ideational originality (Murphy et al., 2013; Runco et al., 2011). Moreover, patients suffering from Parkinson's disease were observed to develop an extraordinary “creative drive” when treated with levodopa and dopamine-agonists (Inzelberg, 2013). Flaherty (2005) proposed that increased dopamine levels lead to heightened baseline arousal and decreased latent inhibition; both of which are characteristic for highly creative individuals (Carson et al., 2003; Martindale, 1999). Thus, a heightened level of dopamine may, by means of “defective” filters for irrelevant information (low latent inhibition), contribute to an overinclusive thinking style — a cognitive basis of creative potential (Eysenck, 1995; Rominger et al., 2011). Schmajuk et al. (2009) put forward a model of how interindividual differences in dopamine-driven novelty-seeking can lead to higher creative potential. Specifically, the authors posit that interactions between subcortical (dopaminergic) and neocortical systems leads to the production of novel associations, and ultimately to creative responses. To sum up, it can be concluded that higher striatal rGMV could be related to higher levels of dopaminergic activity, which may be accompanied by reduced latent inhibition, and an overinclusive thinking style — characteristics typically associated with creativity (Carson et al., 2003; Eysenck, 1995).

### The neural bases of creative potential

Based on the findings presented above, we propose that creative potential might have at least two distinct and functionally specific brain structural bases: While the precuneus is known to be part of the DMN and is involved in self-referential thought processes during internally directed attention, the striatum is known to be part of the dopaminergic system and has been linked to novelty-seeking by means of decreased gating of incoming information. Similar to the attentional–associative model proposed by Schmajuk et al. (2009), it can thus be hypothesized that creative potential, firstly, draws on a pronounced exploratory behavior and a lowered gating threshold for external sensory stimuli. In line with this notion, it was found that not just (reduced) latent inhibition, but on a more general level, also openness to experience – the most prominent personality correlate of creative potential – can be associated with dopaminergic genetic variation (DeYoung et al., 2011). Thus, dopaminergic exploration-behavior parallels the idea of openness as an “investment trait” for creativity (Chamorro-Premuzic and Furnham, 2005) that fosters the acquisition of a broad basis of experiences and general knowledge (Ziegler et al., 2012). Secondly, increased precuneus rGMV may be associated with a facilitated internally focused-attention relating to this rich and diverse knowledge. As outlined above, creative people show more pronounced resting-state activity (e.g., Takeuchi et al., 2011), which may reflect a more intense processing of acquired semantic information during internally focused attention. Moreover, creative individuals were found to be better able to switch between “primary” (resting-state) and “secondary” (goal-directed) modes of cognitive processing (Martindale, 1999; Jauk et al., 2012; Vartanian, 2009), thus enabling them to effectively recruit “generative” and “evaluative” modes of thinking (Ellamil et al., 2012) and ultimately come up with original *and* useful ideas.

### The relationship between ideational fluency and brain structure depends on intelligence

The threshold hypothesis posits that an above-average IQ forms a necessary, but not sufficient condition for high creative potential (Guilford, 1967). While recent investigations corroborated the threshold effect on a behavioral level (Jauk et al., 2013; Karwowski and Gralewski, 2013), there is to date only one study on the neurobiological basis of the threshold effect (Jung et al., 2009). In their spectroscopic study, Jung et al. observed different correlations between creative potential and NAA, a marker of neuronal integrity, in groups of lower and higher IQ. We now investigated if there is also a threshold effect in the relationship between creative potential and rGMV. Concerning ideational originality, we did not observe any effects on rGMV in the whole-brain analysis of lower and higher intelligent individuals (which led us to conclude that the effects in the full sample are homogenous across intelligence groups and vanish due to a loss of statistical power^2^). Concerning ideational fluency, in contrary, we observed an effect on rGMV in the lingual gyrus/cuneus exclusively in the lower, but not in the higher intelligent subsample. In other words, ideational fluency is tied to brain structure only in lower, but not in higher intelligent individuals. This result points to a threshold effect of intelligence in the way that fluent production ability is limited by brain structure in lower, but not higher intelligent individuals.

Fink et al. (2014a) also found regional gray matter density in the cuneus to be associated with creative potential. Although considered an early visual processing area, it was observed that the cuneus is not only activated during actual perception but also during visual *imagery* (Kosslyn et al., 2001). Given that visual imagery is known to be an important strategy during divergent thinking (Gilhooly et al., 2007), it might be speculated that fluency in the lower intelligent group might be more strongly tied to visual imagery whereas higher intelligent individuals might use different strategies (that are less closely associated with a specific brain structure) for the fluent production of ideas during divergent thinking. Although more specific functional research needs to be carried out in order to understand the functional role the cuneus might have in divergent thinking, this result fits nicely with our previous investigation on the threshold hypothesis, which showed that the ability to fluently produce ideas (of unknown quality) has a rather low IQ threshold and can thus be considered a minimum requirement of creative potential (Jauk et al., 2013).

Perspectives on creativity that regard the creative process in analogy to evolutionary processes posit that creative idea generation can be understood in terms of blind variation and selective retention (BVSR; Campbell, 1960; see also Jung et al., 2013). That is, creative idea generation is considered a blind variation (BV) process where many ideas of unknown quality are produced in the first stage. In later stages of the production process, only high-quality ideas are selectively retained (SR). To this end, ideational fluency can be thought of as a necessary prerequisite for blind variation. Results from our structural imaging data, now, point to a moderation of this process by the level of intelligence. While fluency might be limited by the brain's “imaginative ability” in lower intelligent individuals, higher intelligent people might also rely on different strategies for the fluent production of ideas.

### Limitations and conclusion

A possible limitation of our study can be seen in the discrepancies of some of our results to those of previous studies: First, while Zhu et al. (2013) observed structural differences in inferior-frontal regions that parallel the findings of functional MRI studies on divergent thinking, we did not observe any prefrontal effects. It has to be noted, however, that the study of Zhu and colleagues is to date the only VBM study that reported *structural* differences in these prefrontal regions (although there is strong evidence for functional contributions of prefrontal regions; see Gonen-Yaacovi et al., 2013). This might be due to the employed overall creativity score (that does not account for distinct facets of creative potential such as fluency and originality) and/or the procedure used in VBM analysis (Zhu et al., 2013). Second, although the present results are remarkably similar to a previous study from our lab (Fink et al., 2014a), Fink et al. found correlates of originality in the cuneus and combined fluency/flexibility in the precuneus. Concerning these facets of creative potential, the effects observed here seem to be just the other way around. To this end, it should be noted that the creative potential scores used by Fink et al. were substantially correlated (possibly also leading to the overlap in regional gray matter density effects), while the scoring method employed here yielded almost orthogonal scores. Moreover, we used latent variable modeling that can effectively account for measurement error in observed variables (which, together with the non-student sample that was not restricted in intelligence variance, might also explain the high correlation between originality and intelligence; see also Jauk et al., 2014). Nonetheless, the discrepancies remain somewhat puzzling, and only further investigations focusing explicitly on the facets of creative potential (rather than using composite scores) will help to clarify the situation. Moreover, future morphometric studies of creative potential could benefit from including additional structural parameters (such as cortical thickness; cf. Jung et al., 2010).

A recent study describes sex differences in the relationship of brain structure (i.e., white matter connectivity) and creative potential (Ryman et al., 2014). Therefore, we performed a brief post-hoc examination of whether the main results of the present study might also be moderated by sex. We conducted sex-split multiple regression analyses for the prediction of rGMV in the caudate nucleus and the precuneus. While effects of ideational originality on caudate rGMV were significant in both sexes, the effect in the precuneus was only significant in the female subsample. Although a formal test of interaction (sex*originality) was not significant, this result suggests that associations between precuneus rGMV and ideational originality tend to be more strongly driven by the female population. While the finding should be interpreted with great care (because of the rather small male subsample in this study), it points to the importance to consider sex as an important factor in future studies.

The choice of the method used to correct for multiple comparisons is a crucial question in MRI research (Woo et al., 2014). We corrected for multiple comparisons by means of 3dClustSim, which estimates a random field of noise in a given voxel space that serves as the null distribution for significance tests. By this means, it is possible to obtain the minimum size of (true positive, non-noise) clusters at given voxelwise (*p* < .001) and cluster-level (*p* < .05) thresholds. It has recently been suggested, however, that simulation-based correction methods have shortcomings in dealing with the non-stationarity of real brain images, which might lead to false positive results (Silver et al., 2011). As an alternative correction method, threshold-free cluster enhancement (TFCE) was proposed as it is based on permutation tests using real data and shows high sensitivity to both localized peaks as well as spatially extended clusters of low intensity (Smith and Nichols, 2009). Thus, we additionally examined the effect of TFCE correction to our main analysis (effects of creative potential; full sample).^3^ The obtained results pattern was similar, but for TFCE not significant (clusters in precuneus and striatum were only observed at FWE-corrected *p*-values of .08 for the precuneus, .12 for the striatum). We conclude that the TFCE method seems to be more conservative. It should be noted, however, that the precuneus effect was previously reported in three out of four VBM studies of verbal creative potential. This consistency of findings strongly suggests that this finding represents a true positive result (in terms of signal detection).

This study investigated brain structural correlates of different facets of creative potential. We used latent measures of ideational fluency and ideational originality considering the influence of intelligence and openness. We found that originality draws upon regional gray matter volume in striatal regions as well as in the precuneus. The association with striatal regions might point to the role of the dopaminergic system in creative cognition, which foster exploratory behavior in terms of novelty-seeking and might thus lead to the development of a large corpus of experiences and knowledge. The association with the precuneus may be related to a higher proneness toward internally directed attention and mental simulation processes in creative individuals. Moreover, we observed a threshold effect in the relationship between ideational fluency and cuneus rGMV: Fluency was tied to brain structure only in lower, but not in higher intelligent individuals. Taken together, the main findings of this study seem to be remarkably consistent with previous research and provide encouraging perspectives on the biological bases of creative potential.

## Figures and Tables

**Table 1:** Descriptive statistics and intercorrelations.

	Min	Max	*M* (*SD*)	2	3	4	5	6	7	8	9	10	11
CP: Originality (1)	− 0.25	0.26	0.03 (0.11)	.17	.70	.29	.40	.20	.18	.33	− .18	.29	− .16
CP: Fluency (2)	− 4.04	8.15	0.15 (2.34)		.08	.30	.10	.06	.16	.11	.12	.03	.14
Intelligence (3)	− 1.14	1.70	0.09 (0.59)			.11	.33	.10	.13	.24	− .19	.17	− .14
Openness (4)	− 10.92	9.07	0.42 (4.25)				.07	− .02	.05	.04	− .02	− .10	.03
GM (5)	511.30	882.89	666.18 (74.97)					.63	.29	.86	− .25	.52	− .05
WM (6)	421.72	712.21	565.37 (69.80)						.60	.91	.25	.62	.02
CSF (7)	141.80	296.95	207.64 (33.68)							.65	.50	.46	.11
TIV (8)	1129.44	1799.51	1439.20 (148.82)								.11	.65	.01
Age (9)	18.08	55.67	28.42 (10.24)									.18	.38
Sex (10)	1.00	2.00	1.38 (0.49)										.07
Scanner (11)	1.00	2.00	1.70 (0.46)										

Note. *N* = 135. Correlation coefficients above *r* = .17 are significant at *p* < .05, coefficients exceeding *r* = .22 are significant at *p* < .01. CP = Creative Potential, GM = Gray Matter, WM = White Matter, CSF = Corticospinal Fluid, TIV = Total intracranial Volume (GM + WM + CSF).

**Table 2:** Standardized effects of the study variables on rGMV in the precuneus and striatum, seperate for lower and higher IQ subsamples.

	Precuneus				Striatum			
	Lower IQ		Higher IQ		Lower IQ		Higher IQ	
	β	*p*	β	*p*	β	*p*	β	*p*
CP: Originality	0.39	0.00	0.40	0.01	0.46	0.00	0.38	0.01
CP: Fluency	0.28	0.01	− 0.05	0.69	0.04	0.75	0.02	0.86
Intelligence	− 0.23	0.07	− 0.24	0.05	− 0.22	0.10	− 0.09	0.50
Openness	− 0.24	0.04	− 0.28	0.03	0.09	0.44	− 0.12	0.38
Age	− 0.41	0.00	− 0.40	0.00	− 0.31	0.02	− 0.10	0.42
Sex	− 0.07	0.57	− 0.14	0.25	− 0.15	0.24	− 0.24	0.06
Scanner	− 0.07	0.58	0.12	0.31	0.14	0.29	0.33	0.01

Note. *N*_lower IQ_ = 67, *N*_higher IQ_ = 67. CP = Creative Potential.

## Footnotes

1 The participant pool did not overlap with the previous VBM study from our lab (Fink et al., 2014a).

2 This view is further supported by our complemental ROI-analyses in the precuneus, where beta-weights stayed virtually equal across groups of lower and higher IQ (see Table 2).

3 The algorithm used was developed for SPM by C. Gaser/University of Jena and is available online at http://dbm.neuro.uni-jena.de/tfce/.
